# Antibody levels against BK virus and prostate, kidney and bladder cancers in the EPIC-Oxford cohort

**DOI:** 10.1038/sj.bjc.6602869

**Published:** 2005-11-22

**Authors:** R Newton, T Ribeiro, D Casabonne, E Alvarez, A Touzé, T Key, P Coursaget

**Affiliations:** 1 Epidemiology & Genetics Unit, Department of Health Sciences, Area 3, Seebohm Rowntree Building, Heslington, York YO10 5DD, UK; 2 INSERM U618, Faculté de Pharmacie, 37200 Tours, France; 3 Cancer Epidemiology Unit, Richard Doll Building, University of Oxford, Old Road Campus, Roosevelt Drive, Headington, Oxford OX3 7LF, UK

**Keywords:** BK virus, prostate cancer, EPIC

## Abstract

In a case–control study nested within the EPIC-Oxford cohort, there were no statistically significant differences in the prevalence or titre of antibodies against BK virus measured in plasma taken prior to diagnosis between cases with cancer of the prostate (*n*=31), kidney (*n*=5) or bladder (*n*=9) and controls (*n*=45).

The BK virus (BKV), a human polyomavirus, is an important cause of nephropathy and graft failure in immunosuppressed renal transplant recipients (Kwak *et al*, 2002). In Western populations, 90–100% of people have antibodies against BKV by the age of 10 years. Latent infection is established in renal epithelial cells and possibly in other tissues. The virus is oncogenic in newborn hamsters and can transform mammalian cells *in vitro*, but there is little consistent evidence of a link with human cancer. In a previous study, the titre of antibodies against BKV was found to be significantly higher among cases with prostate cancer than among controls, although the finding was based on only 11 cases and could have arisen by chance (Newton *et al*, in press). Alternatively, prostate cancer may lead to reactivation of infection resulting in an increase in titre of anti-BKV antibodies. We examined the relation between BK virus antibodies and cancers of the prostate, kidney and bladder, in plasma collected prior to diagnosis, in a case–control study nested within the Oxford component of the European Prospective Investigation into Cancer and Nutrition (EPIC-Oxford).

## MATERIALS AND METHODS

Between 1993 and 1999, 58 000 people aged 20 years and above and living in the UK were recruited into the Oxford component of the European Prospective Investigation into Cancer and Nutrition (for further details of the study see: Riboli and Kaaks, 1997; Davey *et al*, 2003). Recruitment was through collaborating general practitioners, vegetarian and vegan societies, health-food magazines and from friends and relatives of the participants. Data were obtained via questionnaire on demographic, diet and other lifestyle factors and, for 30% of volunteers, a blood sample was taken, sent through the mail to the laboratory, separated, aliquoted and stored in liquid nitrogen. All participants are followed up for mortality and cancer incidence by record linkage with the National Health Service Central Register. Ethical approval was granted by a Multi-Centre Research Ethics Committee.

In this study, cases were people with cancers of the prostate (*n*=31), kidney (*n*=5) or bladder (*n*=9), for whom a plasma sample was available from prior to diagnosis. For each case, one control was randomly selected from among other study participants for whom a plasma sample was available and who had not been diagnosed with cancer, after matching for age, sex, geographical location, length of follow-up and date of blood sampling (±1 month).

Plasma samples were shipped on dry ice to the INSERM U618 laboratory in Tours, France, for BKV testing. Assays were performed by a single investigator (TR), who was blind to the diagnosis of the relevant patients. BKV VLPs were produced in insect cells using recombinant baculovirus encoding BKV VP1 protein, and purified as previously described (Touzé *et al*, 2001). To remove possible background sero-reactivity, sera were also tested against bovine serum albumin (BSA). Flat-bottomed wells of 96-well microplates (Nunc, Life Technologies, Eragny, France) were coated overnight at 4°C with 200 ng of VLPs or BSA in PBS, pH 7.4. After washing with PBS, 0.1% Tween 20 and 200 *μ*l of PBS containing 1% newborn bovine serum (NBS, Sigma, St Quentin Favallier, France) were added (2 h at 37°C). The blocking solution was replaced by 100 *μ*l of sera diluted 1 : 100 in 5 × PBS-10% NBS and 2% Tween 20, and plates were incubated at 45°C for 60 min. After four washes, bound antibodies were detected with a goat anti-human IgG immunoglobulin (diluted 1 : 5000) conjugated to horseradish peroxidase (Sigma). Following incubation at 45°C for 1 h and four washes, 100 *μ*l of a substrate solution containing ortho-phenylenediamine and H_2_O_2_ was added. After 30 min incubation, the reaction was stopped by addition of 100 *μ*l of 4 N H_2_SO_4_, and optical densities (OD) were read at 492 nm. For each serum sample the background reactivity found in the BSA-coated wells was subtracted from the OD found in each of the BKV-VLP coated wells. In addition, titres of antibodies against BKV were assessed by serial two-fold dilution and these are the results that are presented here. On the basis of similar ELISA tests using other recombinant antigens, a result was considered to be seronegative if the titre of antibodies against BKV was below 1 : 100 (Touzé *et al*, 1999).

Statistical analyses were performed using STATA software (version 7.0; Stata Corp. College Station, TX). Odds ratios (ORs) for cancer in relation to anti-BKV antibody titres were estimated in relation to a doubling of titre, with 95% confidence intervals (CI), using conditional logistic regression. Tests for statistical significance of OR were derived from likelihood ratio test statistics. All *P*-values are derived from two-sided tests of statistical significance.

## RESULTS AND DISCUSSION

The mean time between blood sampling and diagnosis of cancer was 3.1 years (standard deviation (s.d.) 1.8 years) for prostate cancer, 2.0 years (s.d. 1.0 years) for renal cancer and 1.4 years (s.d. 1.2 years) for bladder cancer. The prevalence of anti-BKV antibodies was 91% (41/45) among controls and for people with cancers of the prostate, kidney and bladder it was 94% (29/31), 100% (5/5) and 78% (7/9), respectively.

**Figure 1:**
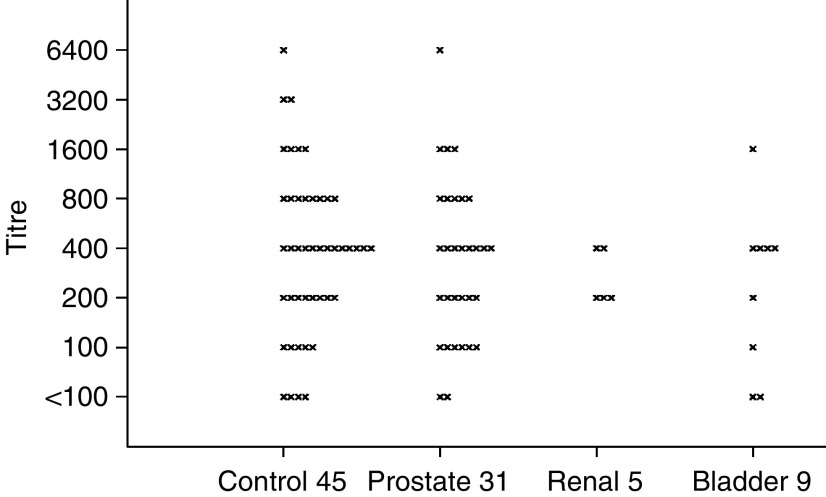
A graph showing the distribution of anti-BKV antibody titres for people with cancers of the prostate, kidney and bladder and for controls in the EPIC-Oxford cohort.

For a doubling of anti-BKV antibody titre, the OR was estimated to change by a multiplicative factor of 0.9 (95% CI 0.6–1.2; *χ*^2^_1_=1.0, *P*=0.3) for prostate cancer (31 cases), 1.8 (95% CI 0.5–7.0; *χ*^2^_1_=0.8, *P*=0.4) for renal cancer (5 cases) and by 0.8 (95% CI 0.4–1.6; *χ*^2^_1_=0.4, *P*=0.5) for bladder cancer (9 cases). Figure 1 shows the titre of antibodies against BKV for each participant both for cases (according to cancer type) and controls.

These results are the first to be reported from a prospective sero-epidemiological study of BK virus in relation to the risk of cancers of the prostate, kidney and bladder. We found no evidence of an association between the prevalence or titre of antibodies against BKV, measured in plasma taken prior to diagnosis, and any of the case groups studied, although the number of people with cancer of the kidney and bladder, in particular, was small. These findings suggest that the association found in a previous study, between high anti-BKV antibody titres and prostate cancer (Newton *et al*, in press), either arose by chance, or resulted from reactivation of infection following tumour development.
